# Proceedings of the Tenth Annual UT-ORNL-KBRIN Bioinformatics Summit 2011

**DOI:** 10.1186/1471-2105-12-S7-A1

**Published:** 2011-08-05

**Authors:** Eric C Rouchka, Robert M Flight, Ramin Homayouni

**Affiliations:** 1 Department of Computer Engineering and Computer Science, University of Louisville, Duthie Center for Engineering, Louisville, KY 40292, USA; 2 Department of Anatomical Sciences and Neurobiology, University of Louisville, Louisville, KY 40292, USA; 3 Department of Biological Sciences, University of Memphis, Memphis, TN 38152, USA

## 

The University of Tennessee (UT), the Oak Ridge National Laboratory (ORNL), and the Kentucky Biomedical Research Infrastructure Network (KBRIN), have collaborated over the past decade to share research and educational expertise in bioinformatics. One result of this collaboration is the joint sponsorship of an annual regional summit to bring together researchers, educators and students who are interested in bioinformatics from a variety of research and educational institutions. This summit provides unique opportunities for collaboration and forging links between members of the various institutions. This year, the Tenth Annual UT-ORNL-KBRIN Bioinformatics Summit was held at the University of Memphis in Memphis, Tennessee from April 1-3, 2011. A total of 225 participants pre-registered for the summit, with 146 from various Tennessee institutions and 60 from various Kentucky institutions. A number of additional participants came from universities and research institutions from other states and countries, e.g. University of Arkansas Medical Sciences, University of British Columbia, University of Cincinnati, Iowa State University, etc. Seventy-four registrants were faculty, with an additional 73 students, 50 staff, and 28 postdoctoral participants.

The conference program consisted of three days of presentations. The first day included a pre-summit of talks by researchers supported by the Kentucky Biomedical Research Infrastructure Network (KBRIN), and three workshops covering the topics Next-Generation Sequencing, the workflow platform Galaxy, and GeneMANIA. The next two days were dedicated to scientific presentations divided into three plenary sessions on Pediatric Genetics, Pharmacogenomics and Systems Biology. Each session also included short talks selected from the submitted poster abstracts.

## Pre-summit KBRIN session

Dr. Eric Rouchka started the pre-summit KBRIN session with an update on the supplement to the primary KBRIN grant awarded in 2009. Thanks to the funding provided by the supplement, five postdoctoral research associates have been hired, along with two masters level bioinformatics staff positions. In addition, a weekly seminar series has been implemented that includes externally invited speakers with strong research programs in bioinformatics.

Following was a series of short talks from various University of Louisville researchers: "Developing an Analysis Pipeline for FT-ICR-MS Isotopologue Data from Stable Isotope Resolved Metabolomics (SIRM) Experiments" (Hunter Moseley), "Time Series Classifier Model for miR-mRNA Relationships" (Jovan D. Rebolledo-Mendez), "Assessing Variations in NGS Data" (Alex Kemper), "A Systems Based Approach to Find Protein Interactions Across Tissues" (Fahim Mohammad), "categoryCompare: High-Throughput Data Meta-Analysis Using Gene Annotations" (Robert M. Flight), "Collaborations between Biology/Bioinformatics" (Benjamin J. Harrison).

## Friday workshops

Jon Armstrong of Cofactor Genomics (St. Louis, MO; http://www.cofactorgenomics.com) started the official summit program with an excellent overview of the past, present and future of DNA sequencing, beginning with the Maxam-Gilbert [1] and Sanger [2] methods and transitioning to the approaches used by next-generation sequencers. Among the technologies covered were pyrosequencing [3], reversible dye terminator technology [4], sequencing by ligation [5], and single molecule real time sequencing [6]. As Jon walked through each of the technologies, he also sought to explain what types of experiments are best suited for each NGS platform, based on the strengths and weaknesses of each technology. Given the rapidly growing number of NGS machines available, the information was extremely useful for both those planning NGS experiments and those analyzing the resulting data.

Following up, Dr. James Taylor from Emory University gave a workshop on the workflow system GALAXY [7,8]. Dr. Taylor started with a comprehensive explanation of the many research issues motivating the development of GALAXY as an easily modifiable, reproducible workflow system for both biologists and bioinformaticians working with high-throughput data. He then proceeded to explain and demonstrate many of GALAXY's features by performing real world analyses using data from the UCSC genome browser [9]. Notable features include the ability to share workflows with others, the ability to generate results pages (similar to full blown publications) with workflows embedded in the document and accessible to anyone viewing the page, and the recent ability for NGS core lab implementations to couple sample requests to workflows so that data processing can occur as soon as the data becomes available. As a final note, Dr. Taylor also demonstrated how easy it is to set up GALAXY instances in Amazons EC2 cloud computing system, enabling one to take advantage of cloud computing systems [10].

The final workshop was given by Dr. Quaid Morris from the University of Toronto on the use of GeneMANIA [11] for pathway and network analysis. GeneMANIA uses gene association networks to assign probable functions of genes based on guilt by association: those genes that share connections (annotations or interactions) probably share other attributes as well. GeneMANIA is available either as a Cytoscape plugin [12,13] or on the web.

## Session I: pediatric genetics

The first session began with opening remarks by Dr. Ramin Homayouni from the University of Memphis. Dr. Homayouni provided a 10 year retrospective of the summit, its beginnings and growth, and the many collaborations and new bioinformatic tools that have resulted from those attending the conference.

The Pediatric Genetics session was truly underway with Dr. Hakon Hakonarson of The Children's Hospital of Philadelphia discussing his research on determining the genetic underpinnings of complex pediatric disorders. As a way to try and cope with the large number of rare genetic variants and their combinations that lead to disease, his group at the Center for Applied Genomics at Children’s Hospital of Philadelphia (CHOP) is targeting to genotype 100,000 children over a five year period making use of a biorepository. One of the goals of this project is to link the genotypes to electronic health care records for the purpose of tying together genotype and phenotype information. Using this data, they have determined SNPs involved in a number of different diseases, including juvenile (Type I) diabetes [14-27], neuroblastoma [28-30] and autism spectrum disorder [31-35]. They hope to be able to use this as a retrospective tool to determine the causes of many other diseases as well.

Following up on that, Dr. Jun Yang from St. Jude Children's Research Hospital presented work on pharmacogenomics and racial disparities in childhood acute lymphoblastic leukaemia [36]. Especially compelling was the ability to use a set of genome wide SNPs to define racial ancestry. These same SNPs also provided a large amount of power in explaining a child’s probability of survival of ALL. In addition to measuring the overall survival probability, Dr. Yang discussed how this information is being used to predict relapse and response to five drugs: DNA, ASP, Chcb, AraC, and 6-TG.

## Session II: pharmacogenomics

Starting the session on pharmacogenomics, Dr. Josh Denny from Vanderbilt University presented work on using electronic medical records for discovery and validation in genome science as part of the eMERGE Network [37]. His group has concentrated on coupling Vanderbilt’s opt-out biobank where samples are genotyped for high-value SNPs, with phenotypes generated through text-mining of associated electronic medical records. Specifically, Dr. Denny has interest in genotyping SNPs in 10,000 samples across 21 loci implicated in atrial fibrulation, Crohn’s disease, type II diabetes, multiple sclerosis, and rheumatoid arthritis. Highlighted challenges in using electronic medical records were presented, included the diagnosis of diseases for which treatment is covered by insurance but which the patient did not have, and the use of templates in doctor’s offices that result in non-informative field descriptions [38]. Both of these proved to be difficult for natural language processing. Dr. Denny discussed case studies of using the Vanderbilt Electronic Systems for Pharmacogenomic Assessment (VESPA) to determine SNPs associated both with disease and pharmacogenomic outcomes [39].

Dr. Ursula Amstutz of the University of British Columbia presented work from the Canadian Pharmacogenomics Network for Drug Safety focusing on reducing drug harm in children [40]. The current process of validating drug safety looks at evidence for drug efficacy and safety at usual doses in populations; however physicians treat individuals, who may have different reactions to a given drug. When adverse drug effects in children are considered, the picture becomes more complicated, due to differences in drug metabolism compared to adults. Working with children who experience cisplatin induced hearing loss, they were able to identify two SNPs that predicted 48% of the cases of hearing loss [41]. Another study examined anthracycline induced cardiotoxicity, finding a highly significant SNP that was validated in a separate group of patients [42]. It is hoped that identification of these SNPs will lead to pre-emptive testing to provide guidance in choosing methods of treatment for children.

## Session III: systems biology

Quaid Morris from the University of Toronto led the final plenary session with a talk titled “Three Degrees of Propagation for Predicting Gene Function Using Networks.” In this presentation, Dr. Morris discussed the idea that biological interaction networks are in general “small world” networks with a high degree of hubs and short paths connecting most nodes. Using the knowledge that often genes in the same pathway are more likely to share neighbors than to be directly connected, gene function can be better predicted from functional gene and interaction networks by considering shared neighbors. Dr. Morris’ group observed that in general, only three to four degrees of propagation provides useful information when considering true gene interactions. After that, every gene in a gene network study is likely to be visited, much in the same way that the idea of “six degrees of separation” is able to link together any two different individuals worldwide within six steps, on average, through “a friend of a friend” relationships [43]. This is the process found in the “Kevin Bacon game” in which participants are challenged to link a random actor or actress to Kevin Bacon in six or fewer steps where two actors/actresses are connected if they appeared in a movie or commercial together [44]. The Morris group has created a new algorithmic approach, 3Prop, which takes this neighborhood information into account to predict gene function. 3Prop extends their previous work of predicting gene function given limited annotation information [11,45]. This approach has been applied to identify the gender of users of social media sites by positively weighting their friends’ genders and negatively weighting their friends’ friends’ genders.

The second talk of the session was the final plenary speaker, David Galas, from the Institute for Systems Biology who presented “Genetics in the Age of Sequencing: Converging on Complexity.” In this talk, Dr. Galas discussed the transformation of human genetics as a result of whole genome sequencing and associated computational approaches. A discussion ensued about the advances that genome wide association studies (GWAS) have led into the understanding of complex diseases [46] , including 1212 published genome-wide associations for 210 traits as of 12/2010 (http://www.genome.gov/gwastudies). However, the point was made that familial-based sequencing can be a powerful technology for complex disease association, as demonstrated by a family of four sequencing project in which two siblings and their parents were sequenced [47]. Dr. Galas discussed the results of this project which was based on two recessive Medalian disorders displayed in the siblings: Miller syndrome and primary ciliary dyskinesia. By looking at familial sequencing, the ability to detect recombination events with high precision is possible, which can lead to more directed analysis of disorders at a genetic level. Dr. Galas expanded upon additional current research at the Institute for Systems Biology, including a multigenerational sequencing project and a project to sequence 600 individuals (all in families) focusing on diseases such as Huntington’s and congenital heart defects.

## Posters and short talks

The poster session was held on day two before the main banquet. Forty-eight posters were on display, all from a variety of different research areas. A number of posters were also selected for short talks in each session. The talks and the presenting authors are listed below for each session. For full author lists and abstracts see the rest of the supplement.

### Session I

"Contribution of common and rare variants in SLCO1B1 to variation in clearance of methotrexate in acute lymphoblastic leukemia patients" (Laura B. Ramsey, St. Jude Children’s Research Hospital), "Significant Analysis of Microarray Using Literature (SAMUL)" (Ramin Homayouni, University of Memphis), "Integrating RNA-Seq data Improves Protein Identification in Shotgun Proteomics" (Xiaojing Wang, Vanderbilt University), "The Challenges and Opportunities Facing us as we Organize Genetic Research Data for "Meaningful Use"" (Ted Kalbfleisch, Intrepid Bioinformatics).

### Session II

"Integrative Biclustering of Heterogeneous Datasets using a Bayesian Nonparametric Model with Application to Chemogenomics" (Dazhuo Li, University of Louisville), "A Statistical Procedure to Evaluate Agreement of Differential Expression for Translational Cross-Species Genomics" (Cuilan Gao, St. Jude Children’s Research Hospital), "Utility of Methylation Biomarkers in Complex Disease" (Gary L Rogers, University of Tennessee - Knoxville), "Targeted Genotyping for Biomarker Development" (Bradford Silver, Computable Genomix).

### Session III

"Identify the Key Genes and Pathways in the Progression of Hepatitis C Virus Induced Hepatocellular Carcinoma Using a Systems Biology Approach" (Siyuan Zheng, Vanderbilt University), "Bioinformatics Analysis of Genome-Wide Differential Gene Expression Patterns Associated with Differential Susceptibility to Severe Group A Streptococcal Sepsis" (Nourtan Abdeltawab, University of Tennessee Health Science Campus), "Genes Without Borders: A Systematic Survey of Mobile Genes and Genomes in Environmental Metagenomes" (Ramy Aziz, San Diego State University), "Design, Implementation and Uses of the Parabiclique Algorithm" (Charles A. Phillips, University of Tennessee - Knoxville).

## Future plans

The 2012 Bioinformatics summit will return to the state of Kentucky in the spring of 2012. Potential focus areas include current technological trends in molecular biology, applications of next-generation sequencing, and systems biology.
